# Antiherpevirus activity of *Artemisia arborescens *essential oil and inhibition of lateral diffusion in Vero cells

**DOI:** 10.1186/1476-0711-6-10

**Published:** 2007-09-26

**Authors:** Manuela Saddi, Adriana Sanna, Filippo Cottiglia, Lorenza Chisu, Laura Casu, Leonardo Bonsignore, Alessandro De Logu

**Affiliations:** 1 Dipartimento di Scienze e Tecnologie Biomediche, Sezione di Microbiologia Medica, Viale Sant'Ignazio 38, 09123 Cagliari, Italy; 2 Dipartimento di Sanità Pubblica, Università di Cagliari, Cagliari, Italy; 3 Dipartimento Farmaco Chimico Tecnologico, Università di Cagliari, Cagliari, Italy; 4 Facoltà di Farmacia, Università di Cagliari, Cagliari, Italy

## Abstract

**Background:**

New prophylactic and therapeutic tools are needed for the treatment of herpes simplex virus infections. Several essential oils have shown to possess antiviral activity *in vitro *against a wide spectrum of viruses.

**Aim:**

The present study was assess to investigate the activities of the essential oil obtained from leaves of *Artemisia arborescens *against HSV-1 and HSV-2

**Methods:**

The cytotoxicity in Vero cells was evaluated by the MTT reduction method. The IC_50 _values were determined by plaque reduction assay. In order to characterize the mechanism of action, yield reduction assay, inhibition of plaque development assay, attachment assay, penetration assay and post-attachment virus neutralization assay were also performed.

**Results:**

The IC_50 _values, determined by plaque reduction assay, were 2.4 and 4.1 μg/ml for HSV-1 and HSV-2, respectively, while the cytotoxicity assay against Vero cells, as determined by the MTT reduction method, showed a CC_50 _value of 132 μg/ml, indicating a CC_50_/IC_50 _ratio of 55 for HSV-1 and 32.2 for HSV-2. The antiviral activity of *A. arborescens *essential oil is principally due to direct virucidal effects. A poor activity determined by yield reduction assay was observed against HSV-1 at higher concentrations when added to cultures of infected cells. No inhibition was observed by attachment assay, penetration assay and post-attachment virus neutralization assay. Furthermore, inhibition of plaque development assay showed that *A. arborescens *essential oil inhibits the lateral diffusion of both HSV-1 and HSV-2.

**Conclusion:**

This study demonstrates the antiviral activity of the essential oil *in toto *obtained from *A. arborescens *against HSV-1 and HSV-2. The mode of action of the essential oil as antiherpesvirus agent seems to be particularly interesting in consideration of its ability to inactivate the virus and to inhibit the cell-to-cell virus diffusion.

## Background

Primary and secondary manifestations of infections sustained by herpes simplex viruses (HSVs) are among the most prevalent human maladies and HSVs are among the wide range of organisms which cause opportunistic infections in patients with AIDS and in patients who are immunosupressed because of other iatrogenic or pathologic reasons, such as organ transplantation or hematologic malignancies [1]. The emergence of drug-resistant strains of HSV, especially in immunosupressed patients, is a major problem and represents a serious concern both in terms of clinical management and of viral ecology [2-4]. Resistance to all major anti-herpetic drugs, such as acyclovir, vidarabine and foscarnet, has been increasingly observed [5-7]. Furthermore, DNA polymerase mutants induced by prolonged or repeated therapy with vidarabine or foscarnet are often resistant also to combination therapy with existing compounds [8-10]. These observations underscore the importance of exploring new and alternative prophylactic and therapeutic tools for the treatment of herpes simplex infections [11,12].

Many plant extracts have been described as potential antiviral agents [13]. Recent reports showed interesting results of antiviral activity of plant extracts in experimental and clinical medicine [14,15] and we have previously demonstrated the antiviral activity in vitro of the essential oil obtained from *Santolina insularis *against HSV-1 and -2 [16].

*Artemisia *species are widespread in nature and are frequently employed for the treatment of several diseases such as malaria, hepatitis, cancer, inflammation and infections sustained by fungi or bacteria [17]. In particular, *A. annua *is known as a remedy for various fevers including malaria [18] and *A. afra*, *A. giraldii *and *A. mexicana *have been described for their antibacterial activity [19-22]. Furthermore, a methanolic extract of *A. caruifolia *was found to inhibit HIV-1 protease and it was demonstrated that this inhibition is due to the presence of tri-p-coumaroylspermidine [23]. The ethanolic and aqueous extracts of *A. arborescens *have been investigated for several biological activities [24], but until now the antiviral properties of the essential oil against HSV-1 and HSV-2 have not been described.

## Methods

### Essential oil

Leaves from *Artemisia arborescens *were collected in Sardinia (Italy), identified and voucher specimens deposited in the herbarium of the Institute of Botany and Botanical Garden, University of Cagliari, Italy. Up to 1500 g of fresh leaves were distilled in a Clevenger-type apparatus for 5 h, the essential oil was dried over anhydrous sodium sulfate and stored at 4°C until use. For the experiments, the oil was dissolved in dimethyl sulfoxide (DMSO) and therefore diluted in the medium. To avoid toxicity or interference by the solvent, the maximum concentration of DMSO in the test medium was 1%.

### Virus and cells

African green monkey kidney cells (Vero) were obtained from the Istituto Zooprofilattico Sperimentale della Lombardia e dell'Emilia (Brescia, Italy). Cells were grown in RPMI 1640 (Gibco) supplemented with 10% fetal calf serum (FCS, Gibco) and penicillin, streptomycin and fungizone (100 U/ml, 100 μg/ml, and 2.5 μg/ml, respectively). Overlay medium for the plaque assays of HSV consisted of Modified Eagle Medium (MEM) without phenol red (Gibco) plus 2% FCS containing antibiotics as described above and 0.5% agarose.

The strains of HSV type 1 (HSV-1 strain F) and HSV type 2 (HSV-2 strain G) used in this study were obtained from the American Type Culture Collection (ATCC), Rockville, Md. HSV-1 and HSV-2 were propagated in Vero cells. Virus titers were determined by plaque assay in Vero cells and are expressed as plaque forming units (PFU)·ml^-1^. The viruses were stored at -70°C until use.

### Cellular toxicity

Cellular toxicity of *A. arborescens *essential oil was tested *in vitro *according to a cell viability assay previously reported [25,26]. Monolayers of Vero cells in 96-multiwell plates were incubated with the essential oil at concentration of 1000 – 15.6 μg/ml in RPMI 1640 for 48 h and the medium replaced with 50 μl of a 1 mg/ml solution of MTT (3-(4,5-dimethylthiazol-2-yl)-2,5-diphenyl tetrazolium bromide, Sigma) in RPMI without phenol red (Sigma). Cells were incubated at 37°C for 3 h, the untransformed MTT removed and 50 μl of acid-isopropanol (HCl 0.04 N in isopropanol) was added to each well. After a few minutes at room temperature to ensure that all crystals were dissolved, the plates were read using an automatic plate reader with a 570 nm test wavelength and a 690 nm reference wavelength.

Maximum Non Toxic Dose (MNTD) was determined microscopically by the observation for morphological changes of cells at 24, 48 and 72 hours of incubation.

### Plaque reduction assay

*A. arborescens *essential oil was first tested for antiviral activity against HSV-1 and HSV-2 by a plaque reduction assay with monolayer cultures of Vero cells grown in RPMI. Cells were infected with 200–250 PFU of HSV-1 or HSV-2. After 1 h adsorption at 37°C plates were washed and medium replaced with MEM containing agarose 0.5%, FCS 2% and different concentrations of essential oil. After 72 h incubation monolayers were fixed with 10% formaldehyde in phosphate buffered saline (PBS), nutrient agar was removed, and cells stained with a 1% solution of crystal violet in methanol 70%.

Some experiments were also performed incubating about 200–250 PFU of HSV-1 and HSV-2 with *A. arborescens *essential oil at concentrations of 100 – 0.19 μg/ml at 37°C or 4°C for varying time periods up to 2 h. Viruses were then adsorbed at 37°C on Vero cells for 1 h, cells were washed and medium replaced with MEM containing agarose 0.5% and FCS 2%. After 72 h incubation at 37°C monolayers were fixed and processed as described above.

The IC_50 _values were calculated by regression analysis of the dose response curves generated from the data.

### Inhibition of plaque development assay

Reduction of plaque development assays were performed as previously described [27] with some modification. Monolayers of Vero cells were infected with about 100 PFU of HSV-1 or HSV-2 for 3 h at 37°C. Cells were then washed and the medium was replaced with nutrient agar containing 100, 50, 25, 12.5 and 6.25 μg of essential oil per ml and 10 μg/ml of HSV-1 and -2 neutralizing antibody (Chemicon International Inc., Temecula, CA) to ensure that plaque development was actually due to cell-to cell virus spread. After 48, 72 and 96 h, the plates were fixed with 10% formaldehyde in PBS for 30 min, the nutrient agar overlay was removed, and the cells were stained with 1% solution of crystal violet in 70% methanol for 30 min. The stained monolayers were then washed and plaque diameter was measured with a digital caliper (Mitutoyo, Japan). Reduction of plaque size by 50% was considered positive inhibition. At least 30 plaques were measured per well. Plaques < 0,2 mm in diameter were considered abortive and therefore were not counted.

### Yield reduction assay

Monolayers of Vero cells grown in 6-well plates were infected by adsorption of HSV-1 or HSV-2 at a multiplicity of infection (MOI) of 1 plaque forming unit per cell (PFU/cell) for 1 h at 37°C. Cells were washed with warm medium and *A. arborescens *essential oil at concentrations ranging between 100 and 3.12 μg/ml in minimum essential medium with 2% FCS was added immediately after adsorption. At 24 h after virus inoculation, cells in the culture medium were lysed by freezing and thawing (three times), and supernatant consisting of culture medium and cell lysate was obtained by centrifugation at 400 × g for 10 min at 4°C. Virus titer was determined by plaque forming assay in Vero cells as described above.

### Attachment assay

Vero monolayers grown in 6-well plates were prechilled at 4°C for 15' and infected with HSV-1 or HSV-2 diluted in serum-free MEM to 200 PFU/ml for varying time periods up to 3.0 h at 4°C in the presence or absence of serial dilutions of *A. arborescens *essential oil (40, 20, 10, 5 and 2.5 μg/ml). Unadsorbed virus was then removed and cells overlaid with nutrient agar. After 72 h cells were fixed and stained as described above.

### Penetration assay

Penetration assays were performed using published procedures with modifications [28]. Briefly, about 200 PFU of HSV-1 or HSV-2 were adsorbed on Vero cells grown on 6-well plates for 3 h at 4°C. The medium was replaced with pre-warmed fresh medium containing *A. arborescens *essential oil (final concentrations 40, 20, 10, 5 and 2.5 μg/ml) and the temperature was abruptly increased to 37°C to maximize penetration of virus. Penetration proceeded for various time period (30 min., 1 h, 1.5 h and 2 h). Monolayers were then treated with PBS, pH 3 for 1 min to neutralize any remaining attached virus and after several washes with serum-free medium cells were overlaid with MEM-0.5% agarose to quantitate surviving virus versus time of essential oil exposure.

### Post-attachment virus neutralization assay

Post-attachment virus neutralization assays were carried out using published procedures with modifications [29,30]. About 250 PFU of HSV-1 and HSV-2 in 0.5 ml of MEM were adsorbed to Vero cells for 2 h at 4°C. Cells were then washed, medium replaced with DMEM containing the essential oil of *A. arborescens *(100 – 12.5 μg/ml) and incubated for 2 h at 4°C. Cell monolayers were again washed and overlaid with DMEM containing 0.5% agarose and incubated at 37° until plaques were fully developed. As a control, HSV-1 and HSV-2 were incubated with serial dilutions of the essential oil for 2 h at 4°C prior to adsorption to cells (pre-attachment neutralization). Cells were fixed and stained as described above, and the number of plaques obtained with control HSV-1 and HSV-2 pretreated with the essential oil was compared with the number of plaques obtained when the essential oil was added after adsorption.

### Antibacterial and antifungal activity

*A. arborescens *essential oil was tested for its antibacterial activity by twofold dilution method in Mueller Hinton Agar (Difco Laboratories) according to standard procedures against five Gram positive (*S. aureus*, *S. epidermidis*, *S. faecalis*, *S. agalactiae *and *B. subtilis*) and six Gram negative species (*E. coli*, *P. aeruginosa*, *K. pneumoniae*, *S. marcescens*, *S. typhi *and *P. mirabilis*) isolated from clinical specimens. The antifungal activity was evaluated against *C. albicans *ATCC E10231 in Sabouraud Dextrose agar. For the evaluation of antimicrobial activity, concentrations of essential oil ranging between 500 and 3.9 μg/ml were employed.

## Results and Discussion

### Cellular toxicity

The CC_50 _of *A. arborescens *essential oil against Vero cells, determined by the MTT reduction assay on confluent monolayers, was 132 μg/ml. The MNTD was determined at 100 mg/ml and this concentration was used as the highest dose in the antiviral assays.

### Antiviral activity

**Figure 1:**
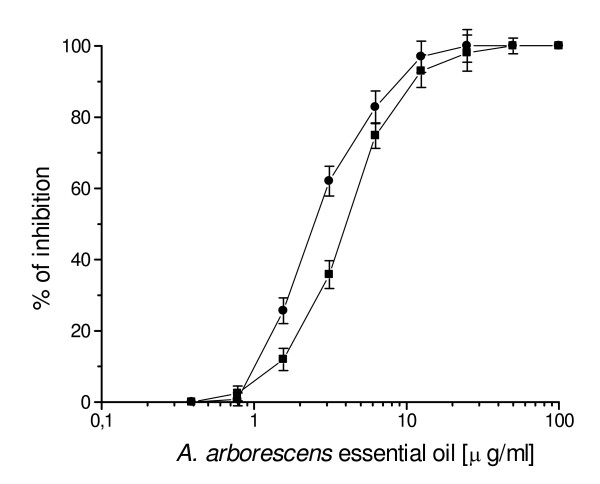
Effects of *A. arborescens *essential oil on plaque formation by HSV-1 and HSV-2. About 250 PFU of HSV-1 (black circle) or HSV-2 (black square) were pre-incubated for 1 h at 37°C in the presence of serial dilutions of essential oil and then adsorbed on Vero cells. *A. arborescens *showed a 50% inhibition of plaque formation respect to the controls at 2.4 μg/ml and a 80% inhibition at 5.6 μg/ml, while against HSV-2 a 50% and 80% were observed at 4.1 μg/ml and 7.3 μg/ml, respectively. The data represent the means for five replicate samples of three separate experiments.

**Figure 2:**
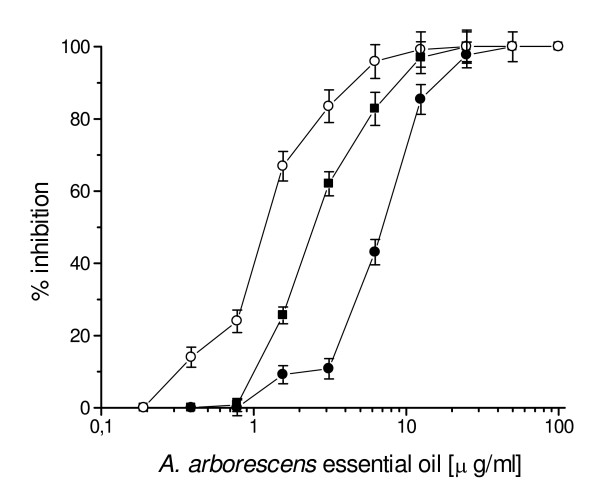
Direct inactivation of different concentrations of *A. arborescens *essential oil on HSV-1 at various times as determined by plaque reduction assay. HSV-1 was mixed with *A. arborescens *essential oil and incubated for 15 min. (black circle), 1 h (black square) or 2 h (white circle) at 37°C. IC_50 _of 2.4 μg/ml as determined after 1 h pre-incubation at 37°C, shifted to 1.14 μg/ml and 6.9 μg/ml when HSV-1 was pre-incubated for 2 h or 15 min, respectively. Results are presented as mean percentage of control of three separate experiments.

**Figure 3:**
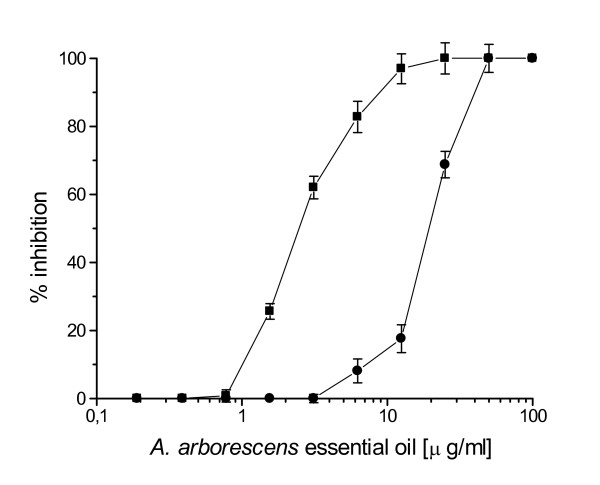
Neutralizing activity of *A. arborescens *essential oil against HSV-1 as determined by plaque reduction assay after 1 h pre-incubation at 37°C (black square) or 4°C (black circle). IC_50 _of 2.4 μg/ml as determined after 1 h pre-incubation at 37°C, increased to 19.4 μg/ml when HSV-1 was pre-incubated for the same time at 4°C. Results are presented as mean percentage of control of four separate experiments.

The activity of *A. arborescens *essential oil against HSV-1 and HSV-2 was first evaluated by a plaque reduction assay. When HSV-1 and HSV-2 were exposed to the essential oil for 1 h at 37°C, *A. arborescens *exhibited a concentration-dependent inhibition of plaque formation compared with the controls (Fig. 1). A 50% inhibition of plaque formation was observed at 2.4 μg/ml and a 80% inhibition at 5.6 μg/ml against HSV-1, while for HSV-2 the 50% and 80% inhibition values were determined at 4.1 and 7.3 μg/ml, respectively. HSV-1 inactivation was clearly dependent on the length of the exposure to the essential oil (Fig. 2), and an higher inhibition was observed when HSV-1 was pre-incubated for 2 h at 37°C (50% and 80% inhibition of plaque formation at 1.14 μg/ml and 2.6 μg/ml, respectively). Furthermore, inactivation was also dependent on the temperature, since pre-incubating HSV-1 for 1 h at 4°C before virus adsorption 50% and 80% inhibition values increased to 19.4 μg/ml and 32.2 μg/ml (Fig. 3).

No inhibition was observed by plaque reduction assay when cells were infected with untreated HSV-1 or HSV-2 and then overlaid with nutrient agar containing essential oil. Furthermore, no inhibition was observed when cells were pre-incubated with the essential oil and then infected with untreated HSV-1 or HSV-2.

### Yield reduction assay

Yield reduction assay showed a dose-dependent antiviral activity of *A. arborescens *essential oil against HSV-2, even if inhibition occurred at concentrations much higher than those needed for neutralization assay. A 70.4% inhibition was observed at a concentration of 100 μg/ml, and a 38.2% inhibition was still observed at 50 μg/ml (data not shown). No antiviral activity against HSV-1 was detected by yield reduction assay.

### Inhibition of plaque development assay

**Figure 4:**
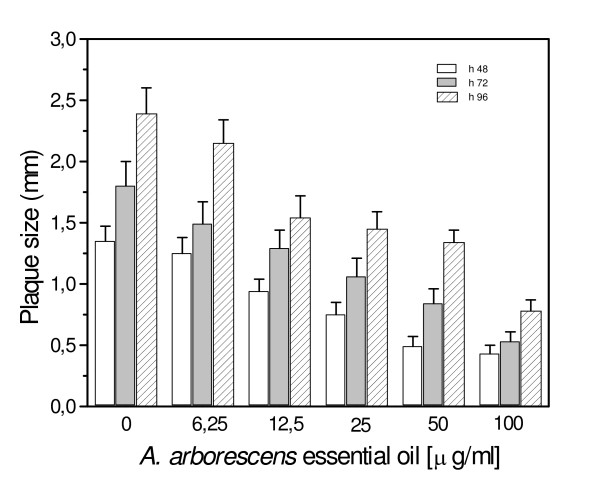
Inhibition of plaque development assay. *A. arborescens *essential oil was applied 3 h post-infection on monolayer of Vero cells infected with 100 PFU of HSV-1. A concentration-dependent reduction of plaque development was observed at 48, 72 and 96 h post-infection. The data represent the means for three replicates of three separate experiments.

**Figure 5:**
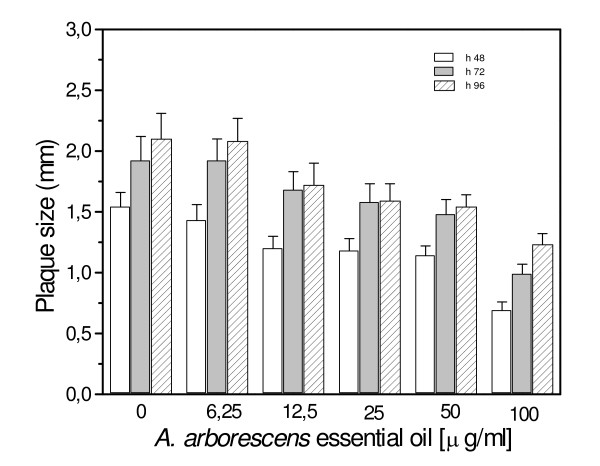
Inhibition of plaque development assay. *A. arborescens *essential oil was applied 3 h post-infection on monolayer of Vero cells infected with 100 PFU of HSV-2. As observed with HSV-1 infected cells, *A. arborescens *induced a concentration-dependent reduction of plaque development at 48, 72 and 96 h post-infection. The data represent the means for three replicates of three separate experiments.

Since it is generally agreed that infectious foci develop when virus infection spreads from infected cells to neighboring uninfected cells, we evaluated the ability of *A. arborescens *to inhibit plaque development when added to cultures of already infected cells. In Figure 4 and Figure 5 are reported results obtained when Vero cells infected with HSV-1 or HSV-2 were overlaid with nutrient agar containing *A. arborescens *essential oil at 100 – 6.25 μg/ml and incubated at 37°C in the presence of neutralizing antibody to ensure that plaque development was actually due to cell-to-cell spread. A concentration-dependent reduction of plaque size was observed for both HSV-1 and HSV-2. In particular, a 68.3% inhibition of HSV-1 lateral diffusion was detected after 96 h incubation in the presence of essential oil 100 μg/ml and a 67.1% inhibition was observed after 48 h incubation with a concentration of 50 μg/ml. A significant reduction of plaque development was shown also by lower concentrations of *A. arborescens*. A statistically significant reduction of plaque diameter was observed for HSV-2, and in particular after 48 h incubation a 55.9% reduction was observed at 100 μg/ml and a 21.7% reduction respect to the untreated controls was still observed at 12.5 μg/ml (Fig. 5).

### Attachment, penetration and post-attachment virus neutralization assays

Virus attachment was inhibited at concentrations of essential oil higher than 50 μg/ml, thus much higher than the doses needed to inactivate the controls of HSV-1 and HSV-2 pre-incubated for 2 h at 4°C in the presence of *A. arborescens*, indicating that attachment was not affected and effects were mainly due to a direct effect on the virion. Furthermore, no inhibition was observed by penetration assay and no inhibition with respect to the controls was detected by post-attachment virus neutralization assay. The number of plaques obtained following adsorption of HSV-1 and HSV-2 in Vero cells at 4°C and incubation, still at 4°C, in the presence of *A. arborescens *was comparable to the number of plaques obtained pre-incubating the viruses alone at 4°C in the presence of the essential oil before virus adsorption (data not shown).

### Antibacterial and antifungal activity

No inhibition of growth of the tested bacteria and *C. albicans *ATCC E10231 was observed at a concentration of *A. arborescens *essential oil of 500 μg/ml after 24 h incubation at 37°C.

## Conclusion

The study has demonstrated the antiviral activity against HSV-1 and HSV-2 of the essential oil *in toto *obtained from *A. arborescens*. Experiments of plaque reduction assay showed a concentration-dependent inhibition of the plaque formation when the viruses were exposed to the essential oil before adsorption with IC_50 _of 2.4 and 4.1 μg/ml for HSV-1 and HSV-2, respectively, therefore at concentrations much lower than the cytotoxic dose for Vero cells (CC_50 _132 μg/ml), indicating a CC_50_/IC_50 _ratio of 55 and 32.2 for HSV-1 and HSV-2. No reduction of the number of plaques was detected when the essential oil was added to monolayers of already infected cells, indicating that antiviral activity of *A. arborescens *is essentially due to direct virucidal effects. Furthermore, virus attachment and penetration were not affected and no effects were observed by post-attachment virus neutralization assay.

Experiments of yield reduction assay indicated that at higher concentrations *A. arborescens *also inhibited the replication of HSV-2 and a significant reduction of cell-to-cell virus spread, as determined by inhibition of plaque development assay, was observed for both HSV-1 and HSV-2. These two aspects are particularly interesting and indicate that the antiviral activity of *A. arborescens *is not only due to direct virucidal effects, but other mechanisms are involved.

The nature and the mechanism of action of the active components of the essential oil is presently unknown and further studies are in progress to isolate the compounds involved in the antiviral activity of *A. arborescens*. Other investigators indicated the presence in extracts of *Artemisia *species of flavones such as 4',6,7-trihydroxy-3',5'-dimethoxyflavone and 5',5-dihydroxy-3',4',8-trimethoxyflavone [19], exiguaflavone A and B [31], artemetin, bonanzin, eupalitin and chrysosplenetin [32]. Flavones have been extensively described for their antiviral activity [33] and it was demonstrated that the anti-HSV-1 activity is not due to the inhibition of virus adsorption, penetration and viral protein synthesis [34], but involves a virucidal activity which results in a prevention of virus adsorption to host cells and subsequent replication. Therefore, flavones might be responsible, at least in part, for the antiviral activity of *A. arborescens*. Since the essential oil showed IC_50 _values against HSV-1 and HSV-2 much lower than IC_50 _showed by flavones tested alone [34,35], it might be supposed that some synergism between the components of the essential oil occurs. Furthermore, it is interesting point out that flavones obtained from other *Artemisia *species showed a significant antibacterial and antifungal activities, while no antimicrobial activity by *A. arborescens *essential oil occurred when tested against bacteria and fungi even at concentrations as high as 500 μg/ml.

Several essential oils have been studied for their activity against HSV-1 and -2 and have been proposed as promising alternative therapeutic tools [13,16,36]. In fact, since their activity is commonly due to a direct virion inactivation, these oils are often effective also against acyclovir-resistant strains [37]. In comparison with already described essential oils, the mode of action of *A. arborescens *essential oil as antiherpesvirus agent is particularly interesting not only in consideration of its ability to inactivate the extracellular virus at concentration much lower than those described for other essential oils, but also for its ability to inhibit the cell-to-cell virus diffusion in already infected cells. Results obtained encourage further investigations in order to isolate and characterize the compound responsible for the antiviral activity of the essential oil.

## Competing interests

The author(s) declare that they have no competing interests.

## Authors' contributions

LC and FC participated in the collection of plant and in the extraction of essential oil. LB participated in the design of the study. AS, MS and LC carried out cellular toxicity studies, antiviral and antibacterial assays. ADL participated in the design, coordination of the study and interpretation of data. All authors read and approved the final manuscript.
